# Therapeutic Potency of Ginger, Garlic, and Pomegranate Extracts Against *Cryptosporidium parvum*-Mediated Gastro-Splenic Damage in Mice

**DOI:** 10.1007/s11686-022-00635-0

**Published:** 2022-11-08

**Authors:** Dina M. M. EL-Shewehy, Gehad E. Elshopakey, Amira Ismail, Shimaa S. Hassan, Amany M. Ramez

**Affiliations:** 1 Zoology Department, Faculty of Science, Mansoura University, Mansoura, 35516 Egypt; 2 Department of Clinical Pathology, Faculty of Veterinary Medicine, Mansoura University, Mansoura, 35516 Egypt; 3 Department of Parasitology, Faculty of Medicine, Mansoura University, Mansoura, 35516 Egypt; 4 Zoology Department, Faculty of Science, Zagazig University, Zagazig, 44519 Egypt

**Keywords:** *Cryptosporidium parvum*, Ginger, Garlic, Pomegranate, P53, Caspase-3

## Abstract

**Purpose:**

*Cryptosporidium parvum* is a protozoan parasite infecting most mammalian hosts and causing major health issues. The present study investigated the efficacy of ginger (*Zingiber officinale*), garlic (*Allium sativum),* and pomegranate (*Punica granatum*) peel extracts on the development and progression of experimental cryptosporidiosis in mice.

**Methods:**

Eighty-two mice were assigned to 6 groups: control, infected non-treated, metronidazole (MTZ), ginger, garlic, and pomegranate. The control group topically received no treatments. The infected non-treated group was experimentally infected by 10^4^ *C. parvum* oocysts per mouse using a stomach tube. The MTZ group was infected with *C. parvum* oocysts combined with MTZ (50 mg/kg b.w./day). The ginger, garlic, and pomegranate groups daily received different plant extracts at doses of 100 mg/kg BW, 50 mg/kg BW, and 3 gm/kg BW, respectively, followed by infection with *C. parvum* oocysts. All treatments were applied orally one day after the infection for continuous 30 days.

**Results:**

Histopathological and immunohistochemical examinations for P53 and caspase-3 expressions in stomach and spleen tissues showed that MTZ and garlic-treated mice had a more significant effect on infected mice.

**Conclusion:**

The garlic extract was found to exert a more pronounced effect on infected mice compared with the other treatments as well as to improve health. Garlic extracts, therefore, represent an effective and natural therapeutic alternative for the treatment of cryptosporidiosis with low side effects and without drug resistance.

## Introduction

*Cryptosporidium parvum* is a globally distributed protozoan parasite that is found in both vertebrates and invertebrates [1]. Infections are transmitted by the fecal–oral route, or through contaminated food or water, and several major waterborne outbreaks have occurred. Outbreaks of zoonotic cryptosporidiosis have been described as major causes of diarrhoeal disease in humans worldwide [2]. Among all identified species of this protozoan, *C. parvum* has been considered an important zoonotic species with a wide epidemiological profile that includes multiple hosts and reservoirs [3]. Contaminated water and food have been blamed for several human cryptosporidiosis outbreaks [4]. Effective therapy for cryptosporidiosis is limited; as a result, the primary form of action will continue to be preventive hygiene management [3]. Although over 200 medicines have been investigated *in vitro* and *in vivo* for their anti-cryptosporidium properties, there is no chemotherapeutic intervention for cryptosporidiosis [5]. Consequently, there is an urgent need for further studies about alternative regimens that can be used against cryptosporidiosis.

Ginger (*Zingiber officinale*, Zingiberaceae) is a commonly used component in a variety of foods and beverages. The active compounds in ginger, such as gingerdoine, gengerdiol, and gingerol, are responsible for its pharmacological effects. Antioxidant, anti-inflammatory, anticancer [6], and antibacterial are some of its pharmacological activities. Further, ginger is previously reported to have anti-parasitic activity against a variety of parasitic infections that are resistant to drugs such as *Toxoplasma gondii, Giardia lamblia, Schistosoma mansoni*, and *Toxocara canis*. However, its effect on *Cryptosporidium* infection is not fully understood.

Another prospective therapeutic agent is garlic (*Allium sativum*), which is a wonderful plant that contains several potentially active chemical compounds. It contains seventeen amino acids, including arginine, as well as at least eight minerals (germanium, calcium, copper, iron, potassium, magnesium, selenium, and zinc), enzymes such as allinase, and vitamins A, B1, and C [7]. Allicin (diallyl thiosulphinate), one of the organosulphate chemicals found in the bulb, is responsible for the physiological activity of dietary garlic*.* It is responsible for the antimicrobial capabilities of fresh garlic as well as its distinct flavor [8]. Garlic has recently been found to provide a variety of health benefits, including antibacterial, antithrombotic, hypolipidemic, and hypoglycemic properties [9]. In addition, garlic has recently become popular as a treatment for intestinal parasitic infections. Its antihelminthic activity has piqued the curiosity of researchers as its administration resulted in a considerable reduction in worm burden, according to their findings [6]. Furthermore, garlic was successfully used to treat cryptosporidiosis and leishmaniasis in 20 AIDS patients in a single uncontrolled study in China [10].

Pomegranate (*Punica granatum*, Punicaceae), sometimes known as the “power fruit,” is a plant that has been utilized in folklore medicine to treat a variety of ailments. It is very common in the Mediterranean region [11]. Pomegranate has a high concentration of antioxidant chemicals that have anti-inflammatory actions [12]. Despite the numerous studies that have been undertaken to investigate the efficacy of pomegranate in the treatment of illnesses and microbial infections [13], much remains unknown about its effects on parasitic infections. Pomegranate extract appears to have beneficial effects in the reduction of intestinal cestodes and trematodes, as well as anti-protozoan activity [14]. Pomegranate peel has recently been described as a promising treatment for *C. parvum* and found to be effective as an anticoccidial as well as an anthelmintic with no side effects [15].

The main aim of this study was to determine the efficacy of ginger, garlic, and pomegranate as alternative and safe treatments for gastro-splenic damage mediated by *C. parvum.*

## Materials and Methods

### Ethics Statement

Animal experiments were reviewed and approved by the Animal Care and Use Committee (MU-ACUC), Mansoura University. The study was performed in strict compliance with the recommendation outlined in the animal ethics procedure and guidelines of Mansoura University. All efforts were made to minimize animal suffering and to reduce the number of animals used in the study.

### Parasite

Department of Parasitology at Theodor Bilharz Research Institute provided *C. parvum* oocysts. The parasite was kept at 4 °C in a 2.5 percent potassium dichromate solution (wt./vol) [16]. The *Cryptosporidium* oocysts were washed three times in distilled water before infection to eliminate potassium dichromate. The oocytes were then centrifuged for 10 min at 1500 g before being counted on a hemocytometer. By diluting the oocyst in the necessary amount of distilled water, approximately 10^4^ oocysts/mL were produced for infection of each mouse [17].

### Experimental Animals

National Research Center, Cairo, Egypt provided male Swiss Albino mice (3–5 weeks), each weighing 25–30 g. The mice were housed in clean, well-ventilated cages with new bedding material every day for one week before the experiment to allow them to adjust to the laboratory environment. To rule out the existence of parasites, the feces of mice were thoroughly investigated using a direct wet salinity method [18]. During the acclimation period, the animals were fed a standard meal and given unlimited water.

### Experimental Design and Sampling

Male Swiss Albino mice (*n* = 82) were randomly assigned to six groups (12 mice/group) as follows: The uninfected control group topically received no treatments. The infected non-treated group was experimentally infected by 10^4^ *C. parvum* oocysts per mouse using a plastic tube (diameter 0.7 mm) attached to a 1 ml syringe. The infected treated with the metronidazole (MTZ) group was infected with *C. parvum* oocysts combined with MTZ (50 mg/kg body weight (BW)/day). The infected treated with ginger, garlic, and pomegranate groups daily received different plant extracts at doses of 100 mg/kg BW, 50 mg/kg BW, and 3 gm/kg BW, respectively, followed by infection with *C. parvum* oocysts. All treatments were applied intragastrical one day after the infection for continuous 30 days.

The animals were slain at the end of the experiment. The stomach and splenic sections were dissected and separated for histological and immunohistological analysis.

### Plant Extract Preparation

#### Ginger

The rhizomes of ginger (*Z. officinale*) were received from the Botany Department, Faculty of Science, Mansoura University, Egypt. The rhizomes were sliced, dried under shade for 7 days, and powdered mechanically using a Moulinex® grinder from France. In total, 100 g of dry ginger powder was added to 400 mL of pure methanol and mixed gently for one hour using a magnetic stirrer. The solution obtained was left at room temperature for 24 h, stirred again, and filtered using filter paper (Grade 1 Whatman cellulose filter papers, Bastone, UK), and the solvent was then removed by evaporation in a rotary evaporator. The residue obtained (4 g) was put into a sterile glass container and stored at 4 °C for further use [19]. The dose chosen for this study was 100 mg/kg BW [20].

#### Garlic

Fresh garlic bulbs were peeled and rinsed in distilled water after being separated. After drying, 500 g of garlic bulbs were ground in a blender until they reached a homogeneous consistency. An aqueous solution with a concentration of 1 g/mL was made by diluting the paste with distilled water. The raw garlic juice was aliquoted and kept at −20 °C until used [21]. Dilution of the stock solution with distilled water yielded the working solution. The dose chosen for this study was 50 mg/kg BW [22].

#### Pomegranate

*P. granatum* peels were obtained from fruit purchased from a local market. The samples were verified by the Botany Department, Faculty of Science, Mansoura University, Egypt. The peels were physically scraped off the fruits, dried in the shade, and then ground into powder in a grinding mill. The methanolic extract of the peel (200 g powder/500 mL methanol) was prepared by mashing in a proportion of 1:2:2 (*w* peel/*v* water/*v* methanol) and left for about 48 h in the fridge. After mashing, the resulting extract was filtered, then the solvent was evaporated under reduced pressure at 40–50 °C and was kept at 4 °C until used [23].

### Phytochemical Analysis

Total flavonoids, cardiac glycoside, total phenols, saponins, tannins, alkaloids, and reducing sugars were quantitatively performed according to previous research [24–30].

### Histopathological Examination

The stomach and spleen tissues were preserved in formalin (10%) before being cut into paraffin-embedded slices. Hematoxylin and eosin (H&E) stain was used to stain the various sections. Histopathological alterations and lesion scores were extensively investigated on the slides [31].

### Immunohistochemical Estimation

To mount on saline-coated glass slides, stomach sections were cut to a thickness of 4 m. After deparaffinized in xylol, the sections were dehydrated using a graded ethanol series. Endogenous peroxidase activity was suppressed with H_2_O_2_ after antigen retrieval (3 percent). After that, tissue sections were treated for one hour at room temperature with primary antibodies against caspase-3 (Lab VisionTM, Int'l: RUO, USA), monoclonal antibody (ready to use), 1:300, and P53 (BioGenex, Milmont Drive Fremont, USA), mouse monoclonal antibody, 1:400. Secondary anti-mouse antibodies were subsequently applied to various slides, and the 3,3′ diaminobenzidine tetrahydrochloride liquid system was used to visualize them.

## Results

### Phytochemical Components of Different Plant Extracts

Table 1 illustrates the phytochemical screenings of plant extracts (mg/g) and shows that the highest active constituents were phenols for ginger, garlic, and pomegranate (18.47, 33.83, and 9.44, respectively); followed by saponins (12.31, 14.56 and 6.32, respectively), cardiac glycosides (11.54, 9.76 and 5.33, respectively), flavonoids (7.21, 17.44 and 5.21, respectively), tannins (1.27, 5.44 and 3.21, respectively) and alkaloids (2.52, 4.32 and 0.24, respectively). The reducing sugar (0.00, 0.94, and 1.53) showed the lowest level of all examined plants.

**Table 1:** Quantitative phytochemical analysis (mean ± SD) of ginger (*Zingiber officinale*), garlic (*Allium sativum*), and pomegranate (*Punica granatum*) extracts

Phytochemicalconstituent (mg/g)	Ginger	Garlic	Pomegranate
Flavonoids	7.21 ± 0.73	17.44 ± 0.73	5.21 ± 1.54
Glycosides	11.54 ± 2.36	9.76 ± 1.46	5.33 ± 1.46
Phenols	18.47 ± 1.52	33.83 ± 3.75	9.44 ± 2.51
Saponins	12.31 ± 2.77	14.56 ± 3.21	6.32 ± 1.22
Tannins	1.27 ± 0.86	5.44 ± 0.52	3.21 ± 0.97
Alkaloids	2.52 ± 0.09	4.32 ± 0.17	0.24 ± 0.05
Reducing Sugar	–	0.94 ± 0.25	1.53 ± 0.34

### Histopathological Finding

#### Stomach

**Fig. 1:**
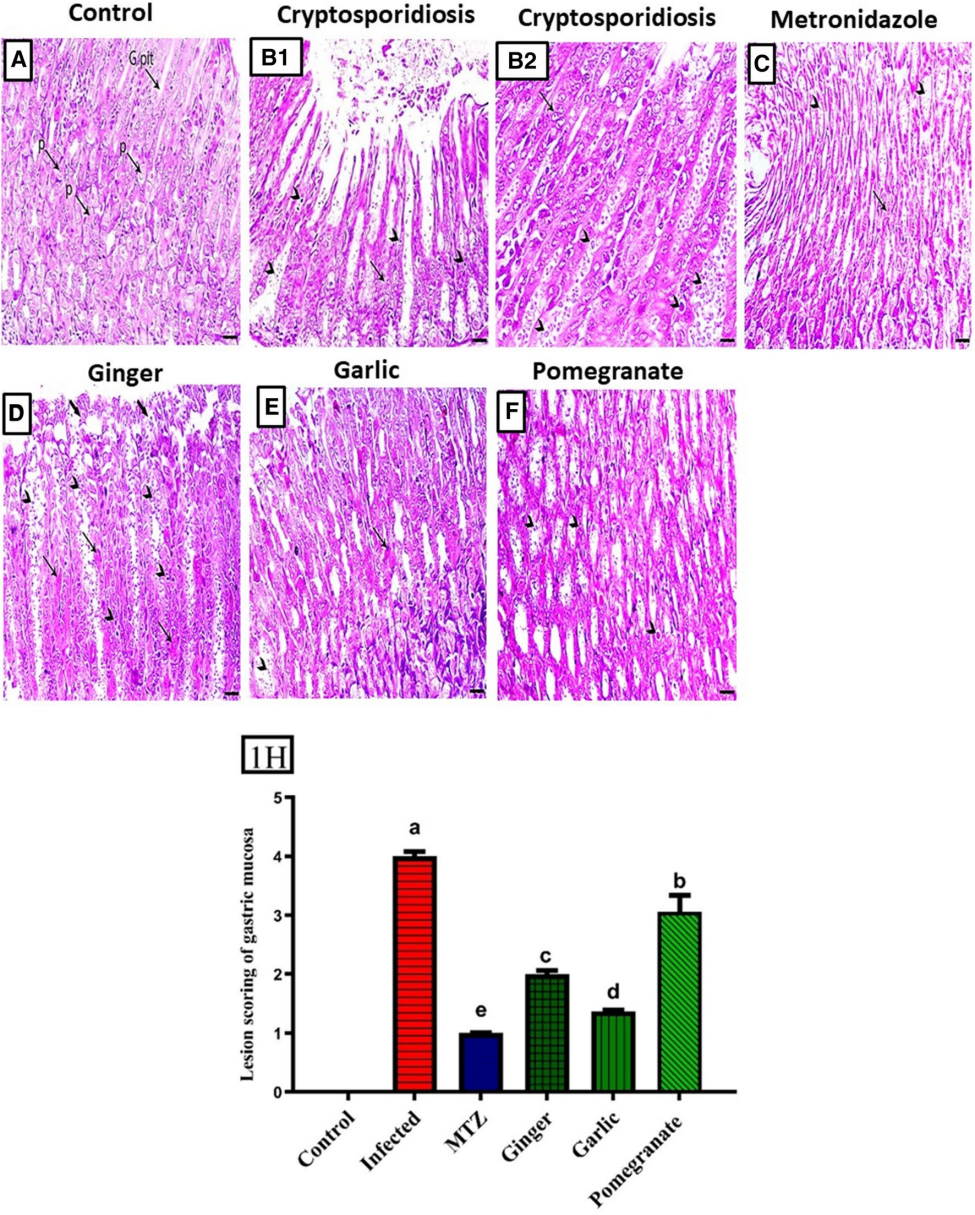
Histopathological sections of gastric mucosa showed: (**A**) Normal control with normal gastric glands. (**B1**, **B2**) Infected control showed marked desquamation of the lining epithelium of the gastric glands associated with the presence of oocysts (arrows head). (**C**) Infected animal treated with MTZ showed mild degenerative lesions of the gastric lining epithelial cells and few oocysts. (**E**) Infected animal treated with ginger showed degenerative changes within the parietal cells with the presence of oocysts within the epithelial cell, (**F**) Infected animal treated with garlic showed mild degenerative lesions of the gastric lining epithelial cells and few oocysts. (**G**) Infected animal treated with pomegranate showed decrease in the epithelial lining degeneration and the presence of oocysts within gastric glands. (1H) Lesion scoring of gastric mucosa in different groups. Data presented as Mean ± SE (*n* = 5), analyzed using one way ANOVA at *p* ≤ 0.05, *significance compared to control

Microscopically, the gastric mucosa of the control group showed normal gastric glands lined by different types of epithelial lining cells including parietal cells and mucous cells lining the gastric pits (Fig. 1A). The gastric mucosa of *Cryptosporidium-*infected animals showed opened and enlarged gastric pits that were filled with necrotic material and mucus, marked desquamation of the lining epithelium of the gastric glands associated with the presence of oocysts either within the epithelial lining or within the lumen of the gastric glands (the changes included the extension of gastric longitudinal folds, epithelial hyperplasia, and mucosal hypertrophy), and marked hyperplastic changes within the mucus cells (Fig. 1B). MTZ and garlic-treated animals showed mild degenerative lesions of the gastric lining epithelial cells and few oocysts (Fig. 1C, E). Furthermore, the gastric mucosa of ginger-treated mice showed desquamation covering mucosa and degenerative changes within the parietal cells associated with the presence of oocysts within the epithelial cell and lumen of the gastric glands (Fig. 1D). A decrease in the epithelial lining degeneration, with still haemosiderin pigment deposition and the presence of oocysts within gastric glands, was recorded in the gastric mucosa of pomegranate-treated mice (Fig. 1F).

#### Spleen

**Fig. 2:**
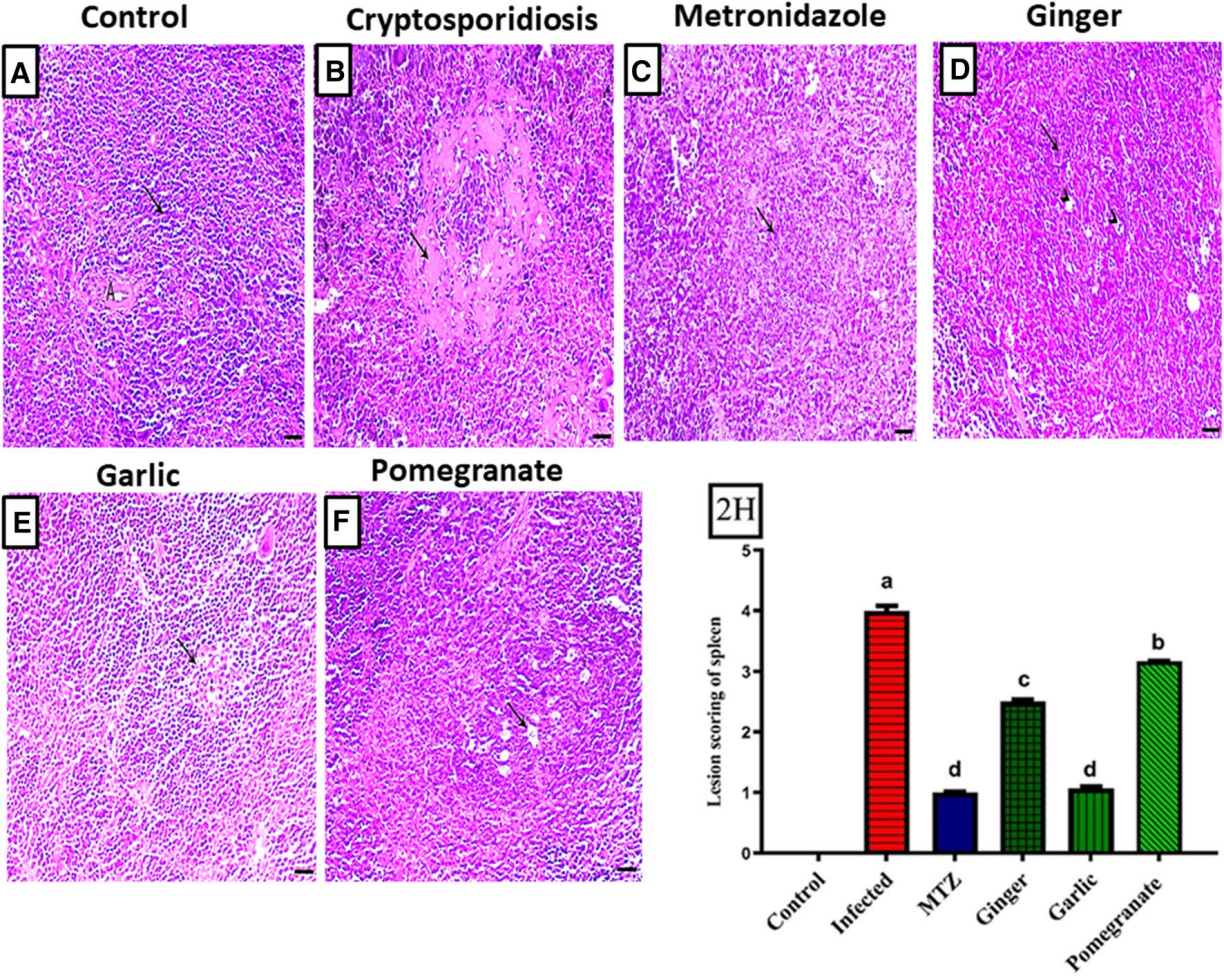
Histopathological sections of spleen showing **A** Spleen of control animal showed normal splenic architecture with normal and white pulps. **B** Spleen of infected untreated mice showed amyloidosis (arrows) associated with severe lymphoid depletion. **C** Spleen of MTZ-treated group showed lymphoid hyperplasia, **D** spleen of ginger-treated mice showed mild to moderate degree of lymphoid depletion, **E** spleen of garlic-treated group showed lymphoid hyperplasia associated with macrophages activation within the follicle, **F** spleen of pomegranate-treated group showed mild lymphoid depletion within the follicle associated with macrophages activation. **2H** Lesion scoring of different groups. Data presented as Mean ± SE (*n* = 5), analyzed using one way ANOVA at *p* ≤ 0.05, *significance compared to control

The spleen of the control animals showed normal lymphoid follicles consisting of lymphocytes around the central arteriole (Fig. 2A). Meanwhile, the spleen of infected mice exhibited interesting amyloidosis (deposition of homogenous eosinophilic material within the white pulp) (extensive hemorrhagic areas) associated with severe lymphoid depletion and splenic vacuolation (Fig. 2B). The spleen of the MTZ-treated group showed lymphoid hyperplasia (Fig. 2C), while that of ginger-treated mice showed a mild-to-moderate degree of lymphoid depletion within the follicle (Fig. 2D). Lymphoid hyperplasia was associated with macrophage activation within the follicle and was demonstrated in the spleen of garlic-treated mice (Fig. 2E). Additionally, the spleen of the pomegranate-treated group demonstrated mild germinal lymphoid depletion within the follicle associated with macrophage activation giving the feature of a starry sky appearance (Fig. 2F).

### Immunohistochemical Findings

**Fig. 3:**
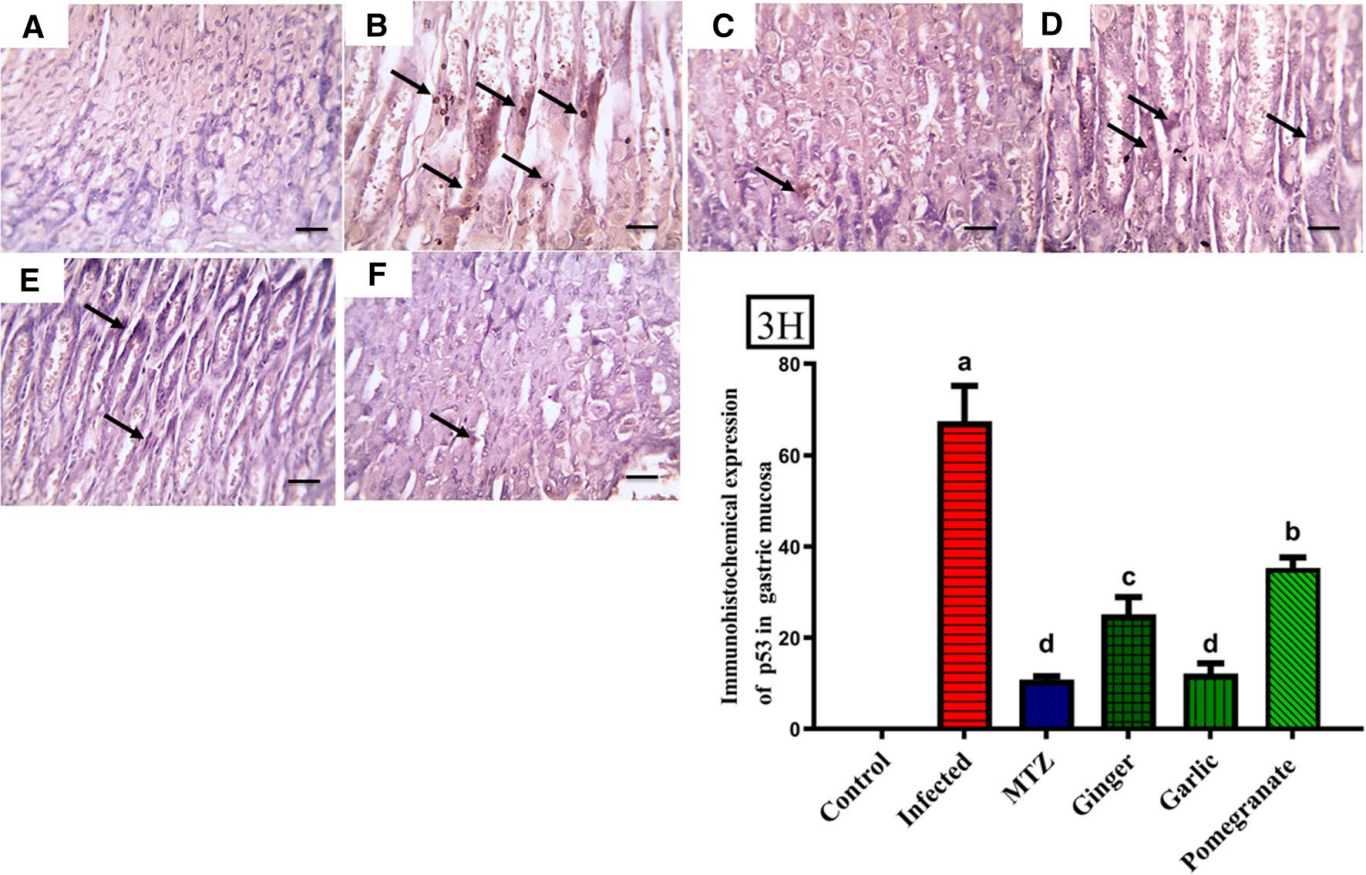
Immunohistochemical sections investigation revealed negative expression of P53 in glandular gastric mucosa of the control group (**A**). Significant positive brown nuclear expression was demonstrated in glandular cells (black arrows) in an infected group (**B**). Markedly decreased positive brown nuclear expression in glandular cells (black arrows) in ginger and pomegranate-treated groups (**D**, **F**), and become weak in glandular cells (black arrows) in MTZ and garlic-treated groups (**C**, **E**). **3H** Quantification of P53 in the gastric mucosa in different groups. Data presented as Mean ± SE (*n* = 5), analyzed using one way ANOVA at *p* ≤ 0.05, *significance compared to control (colour figure online)

**Fig. 4:**
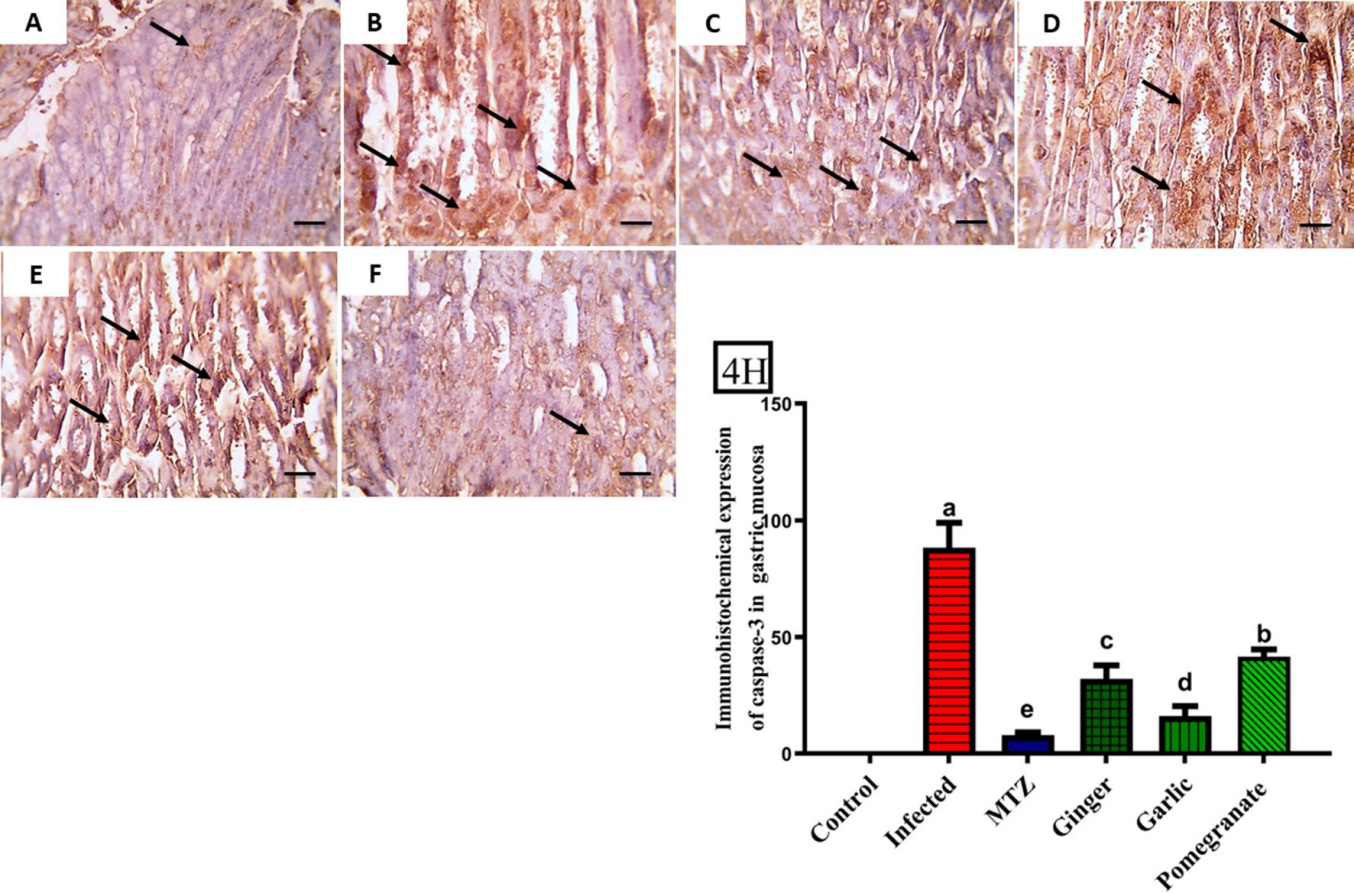
Immunohistochemical sections investigation revealed mild positive brown cytoplasmic expression of caspase-3 was detected in glandular gastric mucosa of the normal control group (**A**). Significantly increased positive brown cytoplasmic expression appears in glandular cells (black arrows) in an infected group (**B**). Markedly decreased positive brown cytoplasmic expression in glandular cells (black arrows) ginger and pomegranate treated groups (**D**, **F**), and become weak in glandular cells (black arrows) in MTZ and garlic treated groups (**C**, **E**). **4H** Quantification of caspase-3 in the gastric mucosa in different groups. Data presented as Mean ± SE (*n* = 5), analyzed using one way ANOVA at *p* ≤ 0.05, *significance compared to control (colour figure online)

Immunohistochemical investigation revealed negative expression of P53 in the glandular gastric mucosa of the control group (Fig. 3A). Significant positive brown nuclear expression was demonstrated in glandular cells (black arrows) in the infected group (Fig. 3B). Markedly decreased positive brown nuclear expression was detected in glandular cells (black arrows) in the ginger- and pomegranate-treated groups (Fig. 3D, F), and the expression become weak in glandular cells (black arrows) in MTZ- and garlic-treated groups (Fig. 3C, E). Additionally, mild positive brown cytoplasmic expression of caspase-3 was detected in the glandular gastric mucosa of the normal control group (Fig. 4A). Significantly increased positive brown cytoplasmic expression appeared in glandular cells (black arrows) in the infected group (Fig. 4B). Markedly decreased positive brown cytoplasmic expression was noticed in glandular cells (black arrows) in ginger- and pomegranate-treated groups (Fig. 4D, F), which become weak in glandular cells (black arrows) in MTZ- and garlic-treated groups (Fig. 4C, E).

## Discussion

*C. parvum* is a widespread parasite and has global significance. Obtaining an efficient therapy for cryptosporidiosis is a high medical demand. Many drugs such as MTZ, are used for the eradication of cryptosporidiosis. However, the resistance of the parasite to such drugs is a growing issue, and thus there is an increasing necessity for the evaluation of new and effective alternative therapies against *Cryptosporidium*.

Previous studies investigated the anti-protozoal activity of garlic, ginger, and pomegranate extracts and their phytochemicals against several protozoal diseases including cryptosporidiosis [32]. However, our study is considered the first to report the protective effects induced by garlic, ginger, and pomegranate against gastric and splenic destruction resulting from the infection by *C. parvum*.

Light microscopic examination of *C. parvum*-infected gastric sections showed desquamation of the gastric glands’ epithelium associated with the presence of oocysts within the epithelial lining and within the lumen of the gastric glands as well as marked hyperplastic changes within the mucus cells [33]. Concerning the gastric sections of the infected MTZ, garlic, ginger, and pomegranate-treated mice, they retrieved their normal structure-like healthy non-infected mice. However, the garlic-treated group showed the greatest enhancement in the shape and structure of the gastric glands compared with other treated mice. These results might be due to the anti-cryptosporidial effect of garlic besides its antioxidant effect that helped the improvement of gastric tissue.

The spleen contains vascular and lymphoid elements, and it is a site of hematopoiesis [34]. The spleen has been less studied in the case of *C. parvum* infection. The inflammatory response was observed in the spleen of mice challenged with *C. parvum* but not harboring the parasite developmental stages. Microscopic examination of *Cryptosporidium*-infected spleen showed atrophy, lymphocyte depletion, and amyloid deposition [35]. While all treated groups showed improvement in spleen histopathology. The best results were observed in the garlic-treated group which showed a noticeable anti-cryptosporidial effect while the other treated groups showed moderate efficiency against *Cryptosporidium*. These results corroborate with previous results of Yang *et al*. who found that the administration of garlic enhances the morphology, weight, and cells of the spleen which exhibited markedly decreased numbers of neoplastic cells [36]. Krishnappa *et al*. observed decreased lymphocytolytic activity, improved congestion, and hemorrhagic areas in the spleen of garlic-treated rats against aflatoxins [37].

Very much like our data, garlic successfully removed the *Cryptosporidium* oocysts from the stool and intestinal sections of the infected immunocompetent mice treated with garlic for two weeks [10]. The garlic oil was also proved to have broad-spectrum antiparasitic activity against certain microorganisms such as *Trypanosoma, Cochlospermum planchonii*, *Leishmania, Plasmodium*, and *Giardia* [38]. The antiprotozoal effect of garlic is due to the presence of several phytochemicals including allicin and several organosulfur compounds such as N-acetylcysteine that possess antimicrobial activity via improving phagocytosis and stimulating the natural killer cells’ activity [39].

Chronic infection with *Cryptosporidium* could hold a chance of inducing gastrointestinal neoplastic alterations. Impedance to apoptosis was a critical step in malignancy progression [40]. *Cryptosporidium* can inhibit the cell death process shortly after infection. The parasite benefits from preventing apoptosis by stabilizing the host cell long enough to enable its life cycle completion [41].

In this study, we observed that the p53 level was upregulated in infected untreated mice whereas it nearly comes to its normal level in all treated groups. However, the best results were obtained in the MTZ and garlic groups. These results agree with Fahmy *et al*. who detected p53 cellular localization in the ileocecal region in *Cryptosporidium*-infected mice. Their results revealed that infection showed focal mild cytosolic p53 labeling in lamina propria compared with non-infected mice in which they did not detect p53 [42]. This showed that *C. parvum* infection exhibited a potential role in the modulation of host cell apoptosis after infection, which gave some additional perception about the impact of cryptosporidiosis on intestinal epithelial growth [43]. Different studies showed that *C. parvum*, which had been isolated from either animals or humans, induced digestive neoplasia in a rodent model [44–46].

In the present study, the presence of caspase-3 in the gastric mucosa was used to detect apoptotic cells. It was low in the control group and present only in a few cells. Enhanced expression of caspase-3 was detected in infected mice. However, the activity of caspase-3 in the treated groups was significantly lower than that in the infected ones. The MTZ and garlic groups displayed weak caspase-3 expression compared with the ginger and pomegranate groups. A similar effect was observed by Abd El-Aal *et al*. who demonstrated that the expression of these apoptotic markers may be required for effective induced immune responses, as they may favor larval destruction and elimination of damaged immune cells that formed during their battle with the parasite [47].

The effects displayed by the garlic derivatives support its effectiveness at protecting against the damage mediated by *C. parvum* infection, indicating the health benefits of garlic such as its antimutagenic, anticarcinogenic, and free-radical scavenging activities as well as its ability to modulate the detoxification systems [48, 49].

## Conclusion

Ginger, garlic, and pomegranate extracts possess anti-cryptosporidial activity (*C. parvum*) that can protect the gastric and splenic epithelia from the damaging effects of *C. parvum* and protect healthy animals from infections. Such results could be adopted in similar infections in susceptible animals and humans**.**

## Data Availability

The datasets generated during and/or analysed during the current study are available from the corresponding author on reasonable request.
